# Rethinking schizophrenia: Optimizing care pathways in Europe from the first episode of psychosis to long-term care

**DOI:** 10.1192/j.eurpsy.2026.10172

**Published:** 2026-02-16

**Authors:** Pavel Mohr, Geert Dom, Peter Falkai, Celso Arango, István Bitter, Julian Beezhold, Patrice Boyer, Ana Catalan, Boris Chaumette, Sonia Dollfus, Cédric Duarte, Paolo Fusar-Poli, Wolfgang Gaebel, Silvana Galderisi, Oliver Howes, Peter Keri, Andreas Meyer-Lindenberg, Merete Nordentoft, Janusz Rybakowski, Jerzy Samochowiec, Agata Szulc, Piotr Toczyski, Joao Vasco Santos, Antonio Vita, Vinciane Quoidbach

**Affiliations:** 1 Third Faculty of Medicine, Charles University, Prague, Czech Republic; 2 National Institute of Mental Health: Narodni ustav dusevniho zdravi, Klecany, Czech Republic; 3 Universiteit Antwerpen, Belgium; 4 Department of Psychiatry and Psychotherapy, LMU: Ludwig-Maximilians-Universitat Munchen, Germany; 5 Hospital Universitario La Paz, Spain; 6 Semmelweis University: Semmelweis Egyetem, Hungary; 7 Suffolk Mental Health Partnership NHS Trust: Norfolk and Suffolk NHS Foundation, UK; 8 Brain Research Foundation, Belgium; 9 University of the Basque Country: Universidad del Pais Vasco, Spain; 10 INSERM U1266: Institut de Psychiatrie et Neurosciences de Paris, France; 11 Caen University: Universite de Caen Normandie, France; 12 Hopitaux Robert Schuman, Luxembourg; 13 King’s College London Section of Cognitive Neuropsychiatry: King’s College London, UK; 14 Heinrich-Heine-Universität Düsseldorf: Heinrich-Heine-Universitat Dusseldorf, Germany; 15 University of Campania Luigi Vanvitelli: Universita degli Studi della Campania Luigi Vanvitelli, Italy; 16 Central Institute of Mental Health: Zentralinstitut fur Seelische Gesundheit, Germany; 17 University of Copenhagen Department of Clinical Medicine: Kobenhavns Universitet, Denmark; 18 Poznan University of Medical Sciences Faculty of Medicine: Uniwersytet Medyczny, Poland; 19 Pomeranian Medical University in Szczecin: Pomorski Uniwersytet Medyczny w Szczecin, Poland; 20 Medical University of Warsaw: Warszawski Uniwersytet Medyczny, Poland; 21 Maria Grzegorzewska Academy of Special Education: Akademia Pedagogiki Specjalnej, Poland; 22 University of Porto Faculty of Medicine: Universidade do Porto Faculdade de Medicina, Portugal; 23 University of Brescia Department of Medical and Surgical Sciences: Universita de Brescia, Italy; 24 University of Warwick – Coventry Campus: University of Warwick, UK

**Keywords:** care pathway, first-episode psychosis, schizophrenia, system of care, early intervention

## Abstract

**Background:**

The European Brain Council project “Rethinking Schizophrenia” aims to examine the existing state of patient care pathways across Europe. The study objectives of Phase 2 were to evaluate the current models of schizophrenia care on mental health outcomes, access and satisfaction with care, and to identify the foundations of integrated care pathways for young people with first-episode psychosis.

**Methods:**

Using the care pathway, a questionnaire was developed and anonymously disseminated to mental health professionals in nine European countries (Belgium, Denmark, France, Germany, Hungary, Italy, Poland, Spain, and the United Kingdom). The survey included 45 questions and addressed the quality and continuity of care from the first onset of psychosis to long-term care, including assessment of the mental health care systems.

**Results:**

A total of 203 responses were obtained. The majority of mental health professionals were psychiatrists (75%), followed by psychologists (11%), nurses (7%), social workers (2%), and GPs and others (5%). The responses were analyzed qualitatively. The results revealed dissatisfaction with the current state of schizophrenia care systems and significant variability in quality of care. The factors that affect the care pathways most are assessment and early intervention services, continuity of care, availability of various types of services and treatments, collaborative decision-making, and utilization of new technologies.

**Conclusions:**

A comprehensive, integrated approach is essential to improve schizophrenia care across Europe. This strategy emphasizes early intervention, equitable access to treatments, coordinated care models, psychosocial support, and long-term recovery through a biopsychosocial framework and emerging technologies.

## Introduction

Schizophrenia is a complex mental disorder associated with substantial morbidity, premature mortality, and profound social and economic burden [1]. Despite advances in pharmacological and psychosocial interventions, many individuals continue to experience poor outcomes, in large part due to gaps across multiple stages of the care pathway. Research has shown that the duration of untreated psychosis (DUP) is a key prognostic factor, with longer delays in treatment associated with worse clinical and functional trajectories [2–4]. Yet, many people face prolonged delays in early symptom recognition, help-seeking, and referral, often compounded by pervasive stigma and discrimination that continue to undermine engagement with services [5, 6].

Even when treatment is initiated, access to evidence-based interventions remains uneven. Coordinated early intervention in psychosis (EIP) services are not universally available, and the provision of recommended psychosocial therapies such as cognitive–behavioral therapy (CBT) for psychosis and family interventions remains sparse [7]. Similarly, pharmacological care is frequently suboptimal: adherence support is inconsistent, long-acting injectable antipsychotics remain underutilized, and clozapine – the gold standard for treatment-resistant schizophrenia – is typically introduced only after years of unsuccessful trials with other agents, despite evidence that clozapine underutilization and delays in its initiation reduce effectiveness and result in poorer outcomes [8–10].

Transitions in care represent another critical vulnerability. The immediate post-discharge period is associated with markedly elevated risks of suicide and readmission, yet follow-up within recommended timeframes is not consistently achieved [11, 12]. Alongside these service gaps, people with schizophrenia experience one of the widest mortality gaps of any major health condition, largely due to preventable cardiometabolic disease, with routine physical health monitoring often failing to meet established standards [7, 13].

Moreover, the wider social determinants of recovery – including housing, employment, and comorbid substance use – are rarely addressed through integrated care, and service users continue to face coercive practices that undermine trust and deter future engagement [5, 14]. Family members and carers, though central to sustaining recovery, are often insufficiently involved in treatment planning and support. Together, these discontinuities highlight not only gaps in clinical provision but also deeper structural shortcomings in service design.

Addressing these issues requires a comprehensive, rights-based, and recovery-oriented approach that integrates early detection, equitable access to evidence-based care, continuity across transitions, proactive management of physical health, and meaningful involvement of service users and carers.

A care pathway is a complex intervention for the mutual decision-making and organization of care processes for a well-defined group of patients during a well-defined period [15]. Schizophrenia care pathways refer to a structured and coordinated series of clinical, psychosocial, and support services designed to guide individuals with schizophrenia through their treatment and recovery journey. The previous European Brain Council (EBC) project “The Value of Treatment” (VoT), conducted in collaboration with the European Psychiatric Association (EPA), the Global Alliance of Mental Illness Advocacy Networks-Europe (GAMIAN-Europe), and the European Federation of Associations of Families of People with Mental Illness (EUFAMI), identified several critical barriers to optimal treatment of people with schizophrenia [16]. The VoT emphasized that early detection and early intervention programs, timely intervention, and relapse prevention are essential for effective management of schizophrenia.

Current mental health care pathways are expected to be diverse across countries and regions. The pathways should be designed so that cross-country/cross-case study comparisons would be possible. The EBC project “Rethinking Schizophrenia,” which is a part of the “Rethinking the Management of Brain Disorders” series, aims to assess the existing state of the patient care pathway across Europe and to offer tangible policy changes to improve the lives of people living with schizophrenia.

In Phase 1 of the project, semi-structured, qualitative interviews were performed with people living with schizophrenia and caregivers, to obtain their insights into how to rethink the way we deal with schizophrenia. The results were summarized in a policy report published in 2024 [17]. Here we present the results from the Phase 2 “Rethinking Schizophrenia” project, which used the findings from Phase 1 to further examine treatment gaps and unmet needs, along with causal factors (patient care pathway analysis from first-episode psychosis to long-term care) in selected European countries.

The study aimed to: (1) evaluate the effectiveness of integrated models of youth mental healthcare in Europe on a broader range of outcomes, including mental health outcomes (clinical symptoms, functioning and quality of life) and health service outcomes; access and satisfaction with care for young people; and (2) identify the common components of integrated care pathways for young people with first-episode psychosis.

## Methods

Using the care pathway as a tool, a questionnaire based on the responses obtained from Phase 1 was developed by the EBC and the EPA in collaboration with a group of schizophrenia experts, including patient and family organizations (GAMIAN-Europe and EUFAMI), and the Czech National Institute of Mental Health.

### Qualitative analysis of open-ended survey responses

The questionnaire included 11 open-ended questions within a 45-item survey designed to capture nuanced, system-level perspectives on gaps and facilitators across the schizophrenia care pathway (e.g., perceived deficiencies in care pathways, barriers in access, challenges in transitions, and examples of positive practice). For the full questionnaire, please see Supplementary Material S1.

Given the exploratory and cross-country health-system focus of the study, free-text responses were analyzed using a qualitative descriptive content approach, combining (1) deductive categorization aligned with the a priori care-pathway domains covered by the survey (early detection/entry, acute care, transitions/continuity, community and social supports, recovery/rehabilitation, and system enablers such as workforce and digital tools), and (2) inductive coding to capture themes not anticipated in advance. This approach is appropriate for producing a pragmatic, policy-relevant synthesis of professionals’ views while staying close to participants’ wording.

### Rationale

A qualitative approach was used because the study addresses complex, context-dependent service and pathway issues that are not fully captured by fixed response options. Open-ended responses allow clinicians and other mental health professionals to explain why barriers occur and how pathways function in practice, supporting an applied synthesis aimed at improving care models across diverse European settings.

Since the care for schizophrenia patients is multidisciplinary, eligible participants were mental health professionals (psychiatrists, psychologists, nurses, and social workers) and general practitioners working in one of the nine participating countries during the survey period. In order to capture perspectives across career stages and settings, a minimum number of years of experience as an inclusion threshold was not applied. “Experience and expertise” were defined pragmatically as professional involvement in schizophrenia/psychosis care pathways, either through: (a) Direct care experience (e.g., assessment, diagnosis, acute treatment, follow-up, psychosocial interventions, and community care) for people with schizophrenia/psychosis (including individuals with first-episode psychosis), and/or (b) System-of-care expertise (e.g., service leadership/management, pathway coordination, guideline development, commissioning, quality improvement, or organization of services relevant to schizophrenia care).

Between 15 May 2024 and 31 August 2024, the survey was disseminated online to experts in nine European countries: Belgium, Denmark, France, Germany, Hungary, Italy, Poland, Spain, and the United Kingdom, through the network of schizophrenia experts in these countries. The platform used to collect data was SurveyMonkey (https://www.surveymonkey.com/r/R59Y5JT). The survey was completed anonymously; the participants were instructed that the responses should reflect primarily the situation in their country, their personal experience, and opinion. The responses were analyzed qualitatively by the expert group and were used to formulate recommendations (both country-specific and at the EU level).

Ethical approval was not required for this study, as it was based on an anonymous online survey in which no identifying information was collected and all questions referred exclusively to systemic and organizational issues, not to sensitive personal data.

## Results

The total of 203 anonymous responses were obtained from nine countries: France, *n* = 46; Hungary, *n* = 35; Italy, *n* = 28; Belgium, *n* = 24; Spain, *n* = 22; Poland, *n* = 19; the United Kingdom, *n* = 10; Denmark, *n* = 7; Germany, *n* = 5; and not disclosed, *n* = 7. The majority of mental health professionals were psychiatrists (75%), followed by psychologists (11%), nurses (7%), social workers (2%), general practitioners (GPs), and others (5%). The responders were 52% female and 48% male. Their mean age was in the range of 46–55 years; 68% of participants had more than 10 years of professional activity, and the mean time of professional experience was between 11 and 25 years. For the full list of sociodemographic and professional characteristics of participants by country, please see Supplementary Material S2: Table 1.

Qualitative data from open questions were summarized to identify factors that facilitated, expedited, or delayed entry or access to the care pathways. The principal findings, shown as the percentage of all respondents, are summarized below.

### Perceptions of current care systems and barriers in access to services

**Figure 1.**
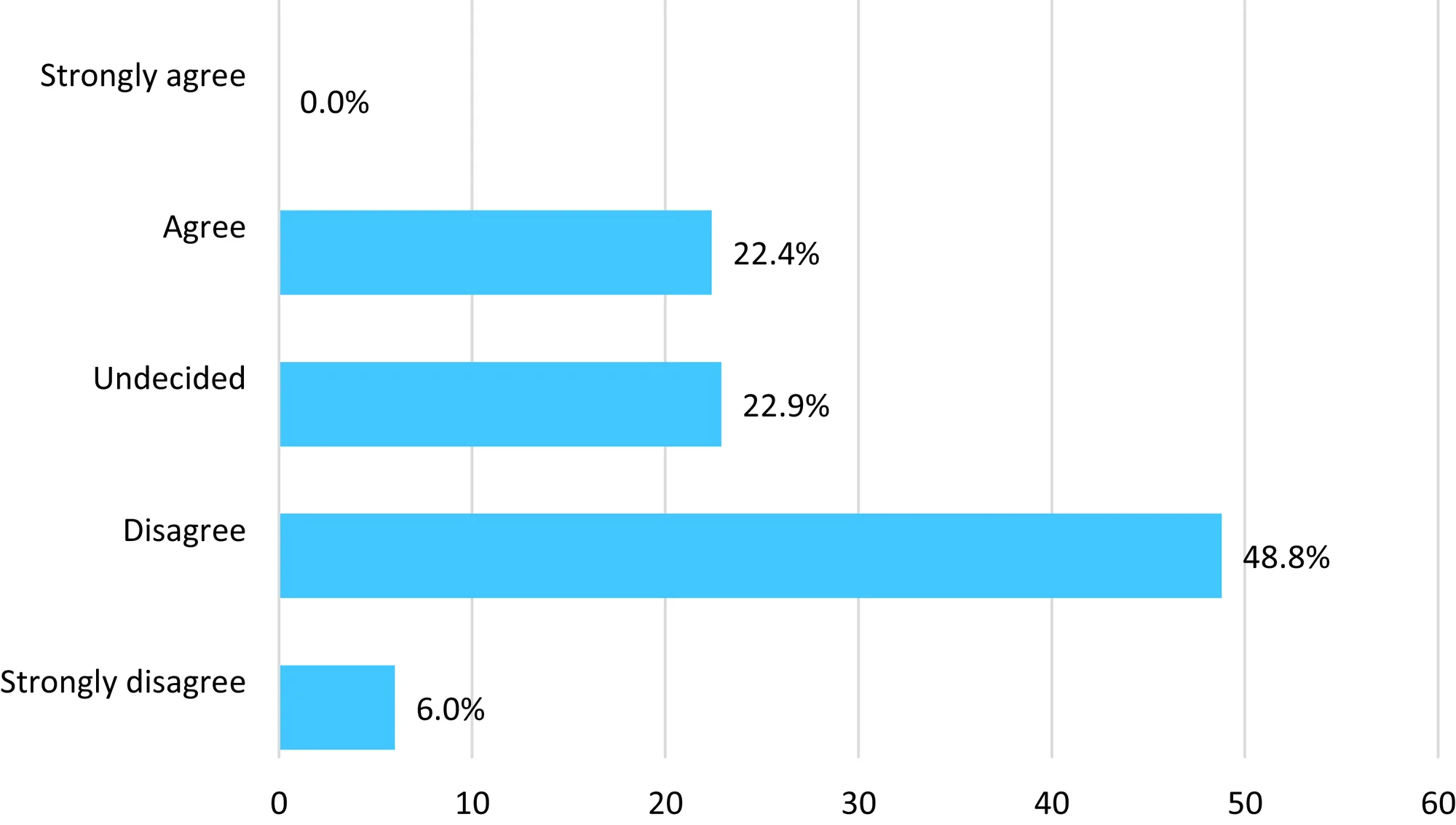
Do you consider the system of care for people with schizophrenia in your country optimal?

The healthcare professionals considered the systems of care for people with schizophrenia in their countries inadequate in more than 50% (Figure 1). They acknowledged numerous deficiencies in the schizophrenia care systems and identified various barriers in access to services, listed in Box 1. Moreover, the majority of the respondents either denied (48%) or were not aware (19%) of any recent (in the past 5 years) anti-stigma program or campaign targeting stigma associated with schizophrenia.

Box 1. Barriers in access to mental health services.

- Long waiting times for appointments and treatment
- Limited outpatient services, particularly early intervention teams
- Poor communication and information sharing between providers, patients, and families
- Lack of equal access to trained therapists
- Coercive strategies of treatment teams
- Nursing should be improved
- Low availability of mental health centers and absence of employment programs
- Geographic inequities (urban vs. rural areas)
- Socioeconomic inequities (high- vs. low-income countries)
- Lack of time and resources, services, facilities, and mental health professionals
- Insufficient use of new services and methods (early detection, prevention, and open dialogue) with overreliance on antipsychotics with side effects
- Delayed crisis intervention
- Discontinuity in care pathway
- Insufficient social support and social care, poor cooperation between medical and social care, and lack of integrated care
- Low availability of psychotherapy and support therapy
- Inadequate care for patients in detention and lack of forensic care programs
- Excessive cost of long-acting injectable antipsychotics (LAIs)
- Discontinuity of care during the transition from child to adult care
- Stigma

### Reforms of mental health systems, community-based mental health care models

**Figure 2.**
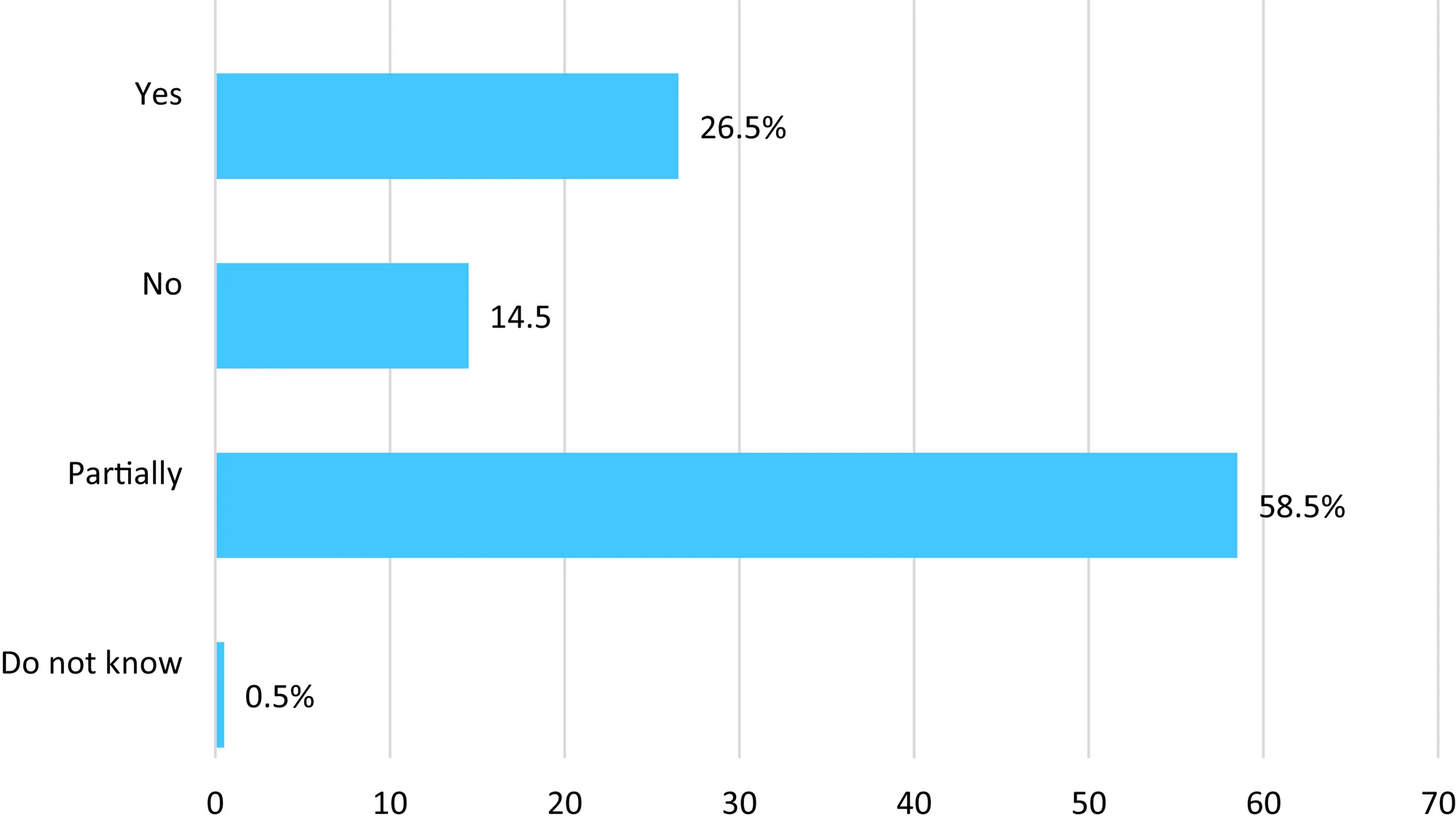
Are the community mental health services widely available?

There are vast inter-country differences in adopting community-based mental health care models and integrated services. One-third of the respondents reported that reforms are progressing too slowly or ineffectively, often due to underfunded systems, workforce shortages, and fragmented policies. Community-based mental health care systems are not or only partially available to 73% of the mental health professionals (Figure 2).

Quotations from the responses to open-ended questions:

“*Community mental health services try to see people in the community rather than in a hospital, but they are underfunded, understaffed, and overwhelmed. They are therefore being pushed to discharge people, some of whom will not be well or having difficulty engaging.*”

“*Community psychiatry is detached from health care, it is a weak social support network with little input from psychiatrists. The reduction of hospital care in the last 25* *years was not followed by an increase in community care and/* *or outpatient services*.”

“*Very different depending on the region. In some of the all the psychiatric hospitals have been closed, whereas in others those still remain but with rehabilitation purposes rather than residential.*”

“*Excellent, with the profiling of an integrated and multidisciplinary community service, oriented towards recovery*.”

### Early assessment and intervention

**Figure 3.**
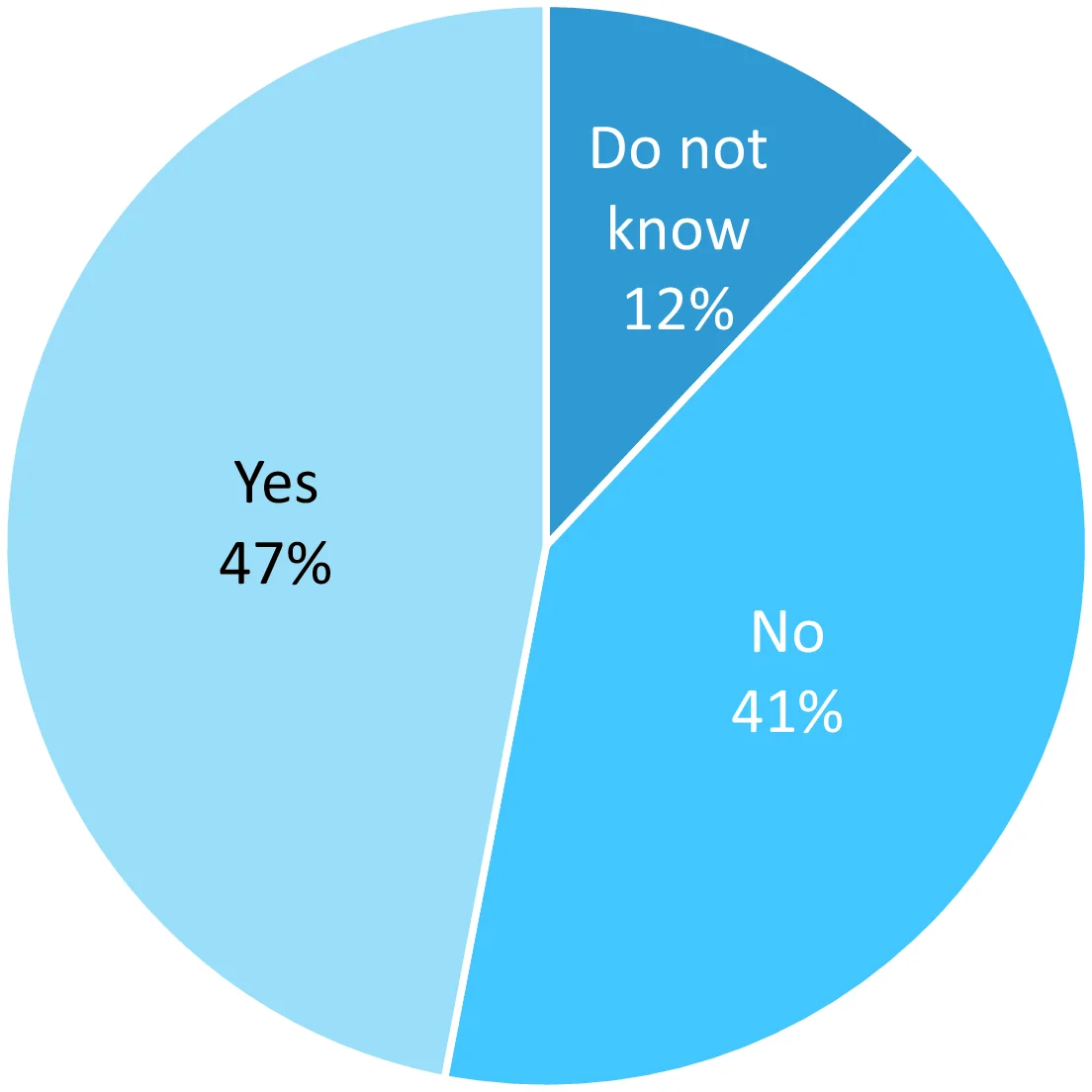
Are there early detection services for at-risk states for psychosis available?

**Figure 4.**
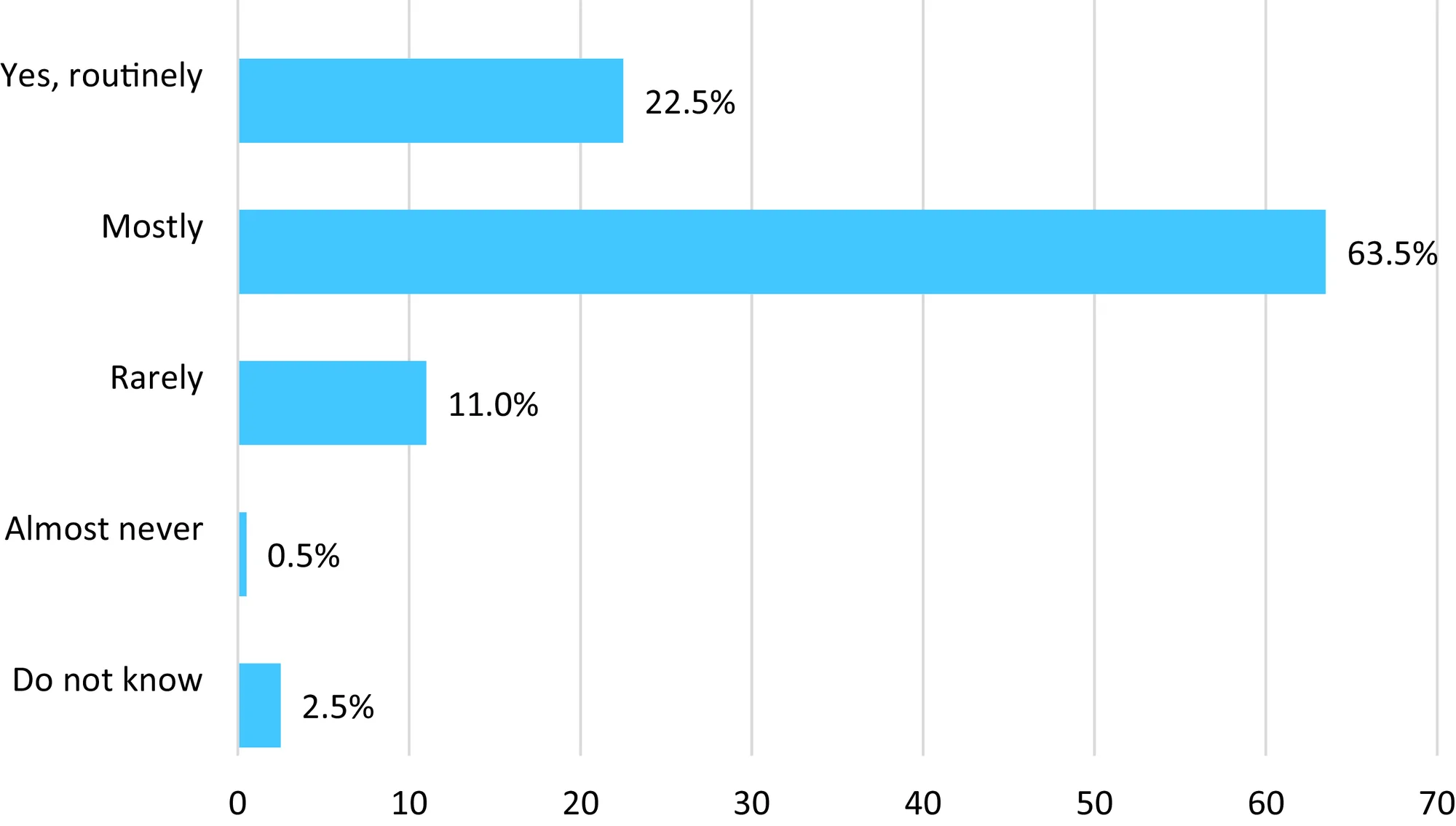
Are people with first-episode schizophrenia hospitalized?

Early detection services are unevenly distributed across the surveyed countries, being available to less than half of the respondents (Figure 3). According to 63% of the mental health professionals, hospital admission for first-episode psychosis (FEP) is often necessary; 52% of participants reported a typical in-patient stay of 2–4 weeks (Figure 4).

### Continuity of care and follow-up care

**Figure 5.**
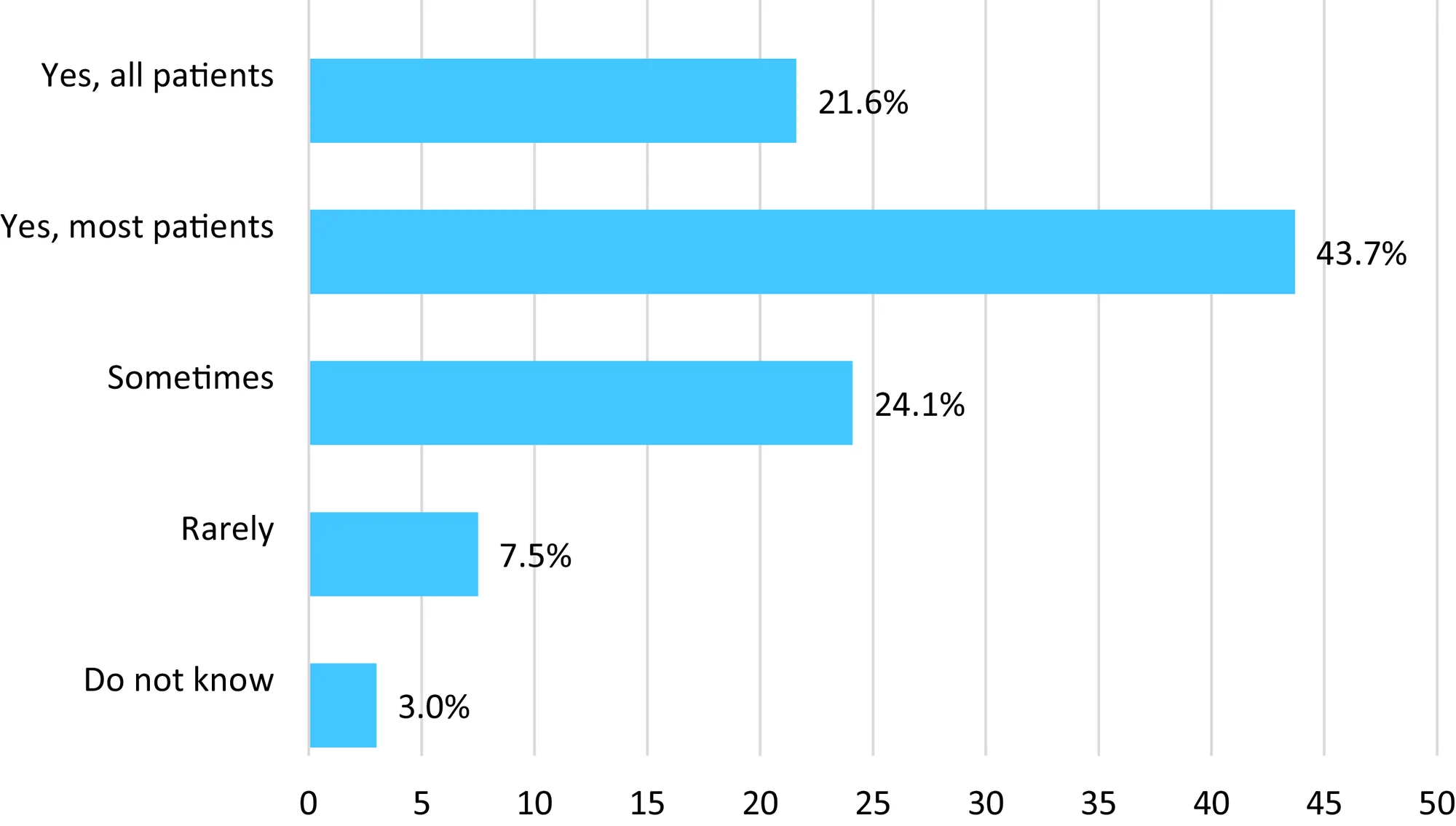
Upon discharge, does the patient have secured continuous treatment and follow-up care?

Survey results revealed gaps in continuous treatment and follow-up care post-discharge. Almost one-third of patients (32%) receive inconsistent post-hospitalization care (Figure 5).

- 59% noted that outpatient care is often not provided by the same therapeutic team
- 81% reported that treatment initiation decision is always or sometimes shared with the patient and carers/relatives
- 73% indicated that family/primary caregivers are always or sometimes involved in the treatment plan
- 22% noted that a collaboration between patients, families, and providers is rare or absent
- 25% reported regular use of multidisciplinary care at treatment initiation
- 55% indicated that the multidisciplinary care is employed selectively
- 28% reported shared responsibilities between general practitioners (GPs) and mental health professionals
- 9% noted that GPs take primary responsibility for integrated care
- 50% noted that day hospitals in their country are partially available
- 63% indicated partial availability of social services.

Quotations from the responses to open-ended questions:

“*Follow* *-up* *care is not systematically organized. Some fall through the cracks or the patient is not willing to have any follow* *-up* *care.*”

“*Lack of resources in the community teams. Pressure to complete 72* *hour follow* *-ups* *post discharge. Poor engagement and inability of services to follow up robustly.*”

“*Finding suitable housing or accommodation. This means patients will sometimes stay in hospital longer than is necessary.*”

“*There is no outreach service able to contact the patients if they do not show* *up.*”

“*It is actually working smoothly, but sometimes capacity in OPUS teams is overburdened.*”

“*Compliance, different doctors prescribe different medication, the patients don’t trust the new, outpatient doctor.*”

### Advancing recovery and functioning

**Figure 6.**
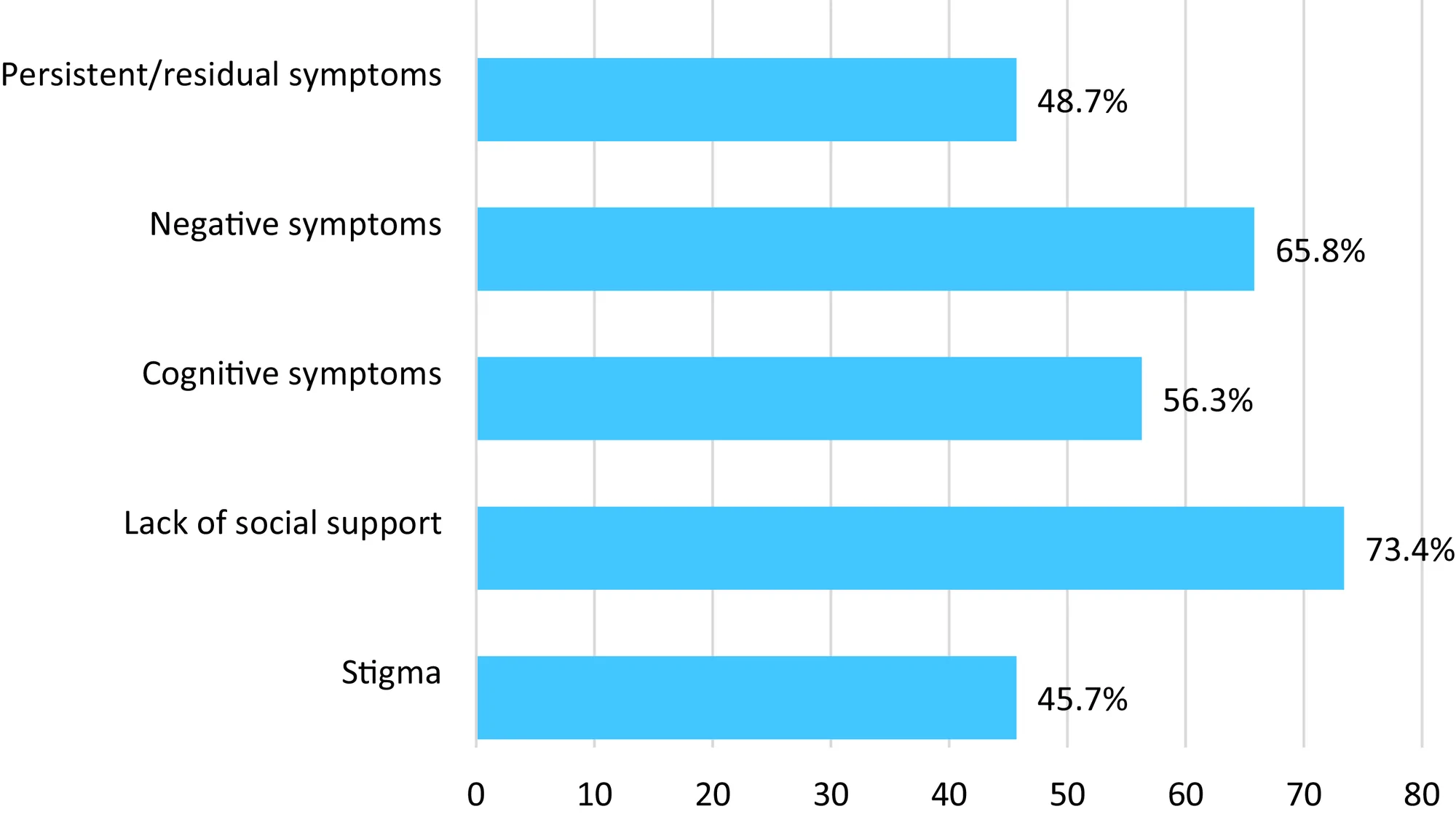
What are the major barriers to full recovery and functioning (possible more than one answer)?

Although negative and cognitive symptoms, together with a lack of social support, are the major barriers to full recovery and functioning (Figure 6), available treatment guidelines primarily rely on accessible antipsychotic medications.

- 68% of participants confirmed the presence of national schizophrenia treatment guidelines
- 54% noted that the guidelines include recommendations for managing both negative and cognitive symptoms
- 14% reported an absence of such guidelines
- 65% of respondents have new antipsychotics registered by the European Medicines Agency (EMA) available
- 14% reported limited access to new antipsychotics
- 82% reported that schizophrenia drug treatments are reimbursed by health insurance
- 25% reported the use of emerging technologies such as Virtual Reality (VR), Artificial Intelligence (AI), and telemedicine.

**Figure 7.**
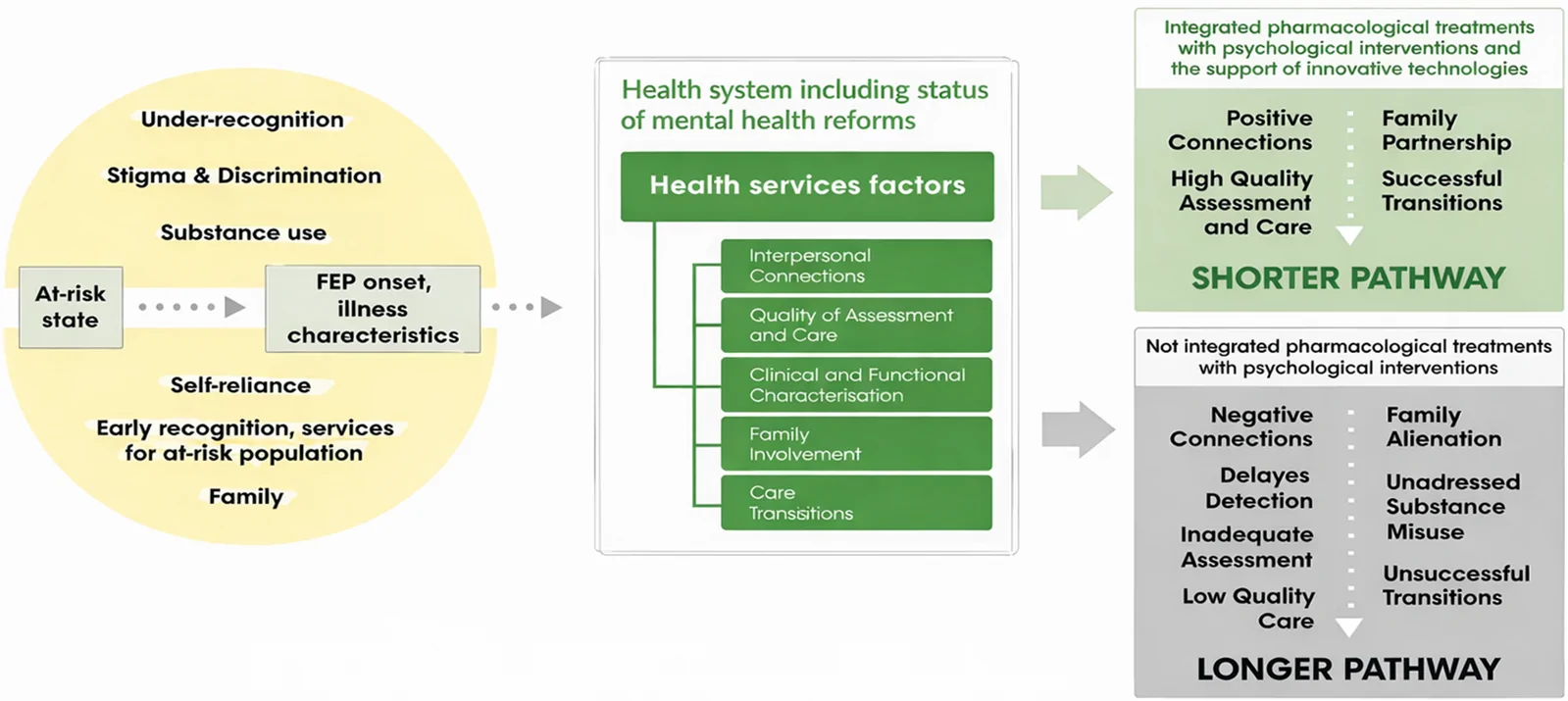
Pathways to care from FEP to long-term care. Figure 7. long description.

Based on the survey results, a clinical model describing the factors that affected the entry or access to the care pathways was developed (Figure 7).

## Discussion

Although the sample size of the participants from all countries surveyed is not representative and the data cannot be generalized, the results provided valuable insights from mental health professionals into the quality and continuity of care from the first onset of psychosis to long-term care across Europe. As the majority of survey participants were psychiatrists and psychologists (86%), more than two-thirds of them with at least 10 years of clinical experience, the findings are grounded in substantial professional expertise. However, despite the effort, input obtained from other groups of mental health professionals directly involved in the care of people with schizophrenia (nurses, social workers, and GPs) is limited. Access to the viewpoints of all stakeholders is essential in better understanding the limitations of the current systems of care.

Most of the studies examining systems of care focused on patients’ unmet needs [18] or economic burden [19]. Other authors studied the patient journey on the national and local level [20, 21]. Our study explored the patient care pathway from first-episode psychosis to long-term care in nine European countries, thus providing a broader perspective. While previous research suggested cultural inter-country differences in the proposed care and interventions [22], we were able to identify several commonly shared elements that affect care pathways. The key factors include assessment and early intervention services, continuity of care, availability of various types of services and treatments, collaborative decision-making, and utilization of new technologies (Figure 7).

Survey results revealed widespread dissatisfaction among the healthcare professionals with the current state of schizophrenia care systems and showed significant variability in the quality of care across different regions of Europe. More than half of the respondents perceived substantial deficiencies in care pathways, accentuating systemic issues, such as fragmented care, delays in assessment and treatment, and lack of access to specialized services. This observation is in agreement with other authors [23, 24].

A long list of barriers hinders access to mental health services across Europe. Among most frequently cited were uneven geographical distribution of the services, long waiting times, delayed access, need to go through the GP’s referral, distrust of pharmacotherapy, coercive and paternalistic approach, stigma, lack of mental health professionals, and low number of outpatient services and early intervention teams. Obstacles in the schizophrenia care pathway may exacerbate disparities in care quality, particularly between socio-economic groups, urban versus rural areas, underlining the need for equitable and patient-centered approaches and the right to treatment [25, 26].

Study participants reported a lack of psychoeducation by healthcare professionals, including physicians, GPs, and nurses. Persisting stigma and discrimination affect individuals with schizophrenia and have a negative impact on education, employment, and social relationships, perpetuating isolation and impeding access to care. Anti-stigma campaigns have shown short-term efficacy in the reduction of discriminatory attitudes, with education-based interventions focused on young people being most effective [5, 27, 28].

Community-based mental health care models are promoted by the EU as the optimal way how to deliver complex care to people with mental illness [29]. While some countries have adopted such models and made progress in integrating services, others continue to struggle with systemic challenges and reforms of psychiatric care. Inconsistent policy implementation across countries limits alignment with international treatment recommendations, algorithms, and guidelines [7, 23, 30].

Early assessment and early intervention services (EIS), multidisciplinary mental health programs that provide comprehensive treatment and support to individuals experiencing their first episode of psychosis or symptom onset, can reduce DUP and the need for hospital admissions, and improve recovery outcomes [4, 23]. Our results indicated that although hospitalization remains a common entry point in FEP, countries with early intervention programs and recovery-oriented care have achieved improved accessibility and engagement [31].

Continuity of care is critical for reducing relapse rates and improving patient outcomes [23]. Transition between care settings, particularly from inpatient to outpatient services, represents a crucial juncture in schizophrenia management. According to the study participants, a lack of coordination and unclear follow-up care options present significant barriers in the transition, often disrupting care. This can lead to treatment discontinuity and poor patient outcomes [12]. Our findings underscore persistent deficiencies in seamless transitions, continuity of care, shared decision-making, multidisciplinary collaboration, and the availability of community-based mental health services. Addressing these issues is essential for advancing patient-centered, recovery-oriented care models. Recovery-oriented care relies on the availability of day hospitals and social services, such as supervised housing and supported employment, including individual placement, as part of the overall continuity process [23, 32]. However, the survey revealed limited access to these resources in many countries.

A multidisciplinary approach enhances care quality by integrating the expertise of various healthcare professionals, including public health professionals and health managers. The role of general practitioners in managing the physical health of individuals with schizophrenia is underutilized. Expanding GPs’ involvement is vital for addressing the physical and mental health comorbidities commonly observed in schizophrenia patients [33, 34]. Shared decision-making, as a cornerstone of personalized mental health care, is still inconsistently practiced. A more inclusive care model that actively involves patients and their families in decision-making is essential for achieving better adherence and satisfaction [35].

Central to the advancements in schizophrenia care is the availability of the second-generation antipsychotics, which offer improved efficacy and tolerability compared to older drugs. However, it is important to acknowledge their limitations: limited efficacy for cognitive and negative symptoms, weight gain, and metabolic side effects. Thus, there is still an unmet need for novel therapeutic agents with an innovative mechanism of action and better tolerability. Encouragingly, 82% of respondents reported that currently available schizophrenia drug treatments are reimbursed by health insurance, ensuring a solid foundation for medication access. Combining pharmacotherapy with psychosocial interventions significantly improves functional outcomes across all stages of schizophrenia [36].

Emerging technologies, such as VR, AI, and telemedicine, remain underutilized, with only 25% of respondents reporting their use. The major barriers in their implementation are the perception of digital technologies as impersonal tools, their questionable credibility, privacy concerns, and absence of knowledge and resources; the key facilitators for their utilization are the usability and accessibility to the patients [37, 38]. These tools already hold promise for enhancing treatment, especially in cognitive rehabilitation and social skills training [39–41]. Furthermore, they have the potential to facilitate early identification and treatment of FEP and at-risk individuals, and to help in monitoring and coordinating service provision. Since digital tools are appealing especially to young people who prefer them to more traditional care delivery, they can facilitate access to youth mental health care [42]. Digital mental health can be also a solution how to overcome logistic and geographical barriers to mental health services, making care more accessible and equitable [43, 44].

There are several limitations of our study. The survey was distributed anonymously through professional networks, so the response rate cannot be calculated. There was a relatively low number of participants and an uneven number of responses across the countries. Moreover, the vast majority of participants were psychiatrists and psychologists; other mental health professions were not sufficiently represented. Therefore, the results are not considered to be representative, with limited generalizability. The questionnaire was disseminated only in English; this fact can pose a selection bias. Importantly, the systems of mental health care differ vastly among European countries; therefore, Phase 3 of the Rethinking Schizophrenia project will now focus on in-depth analysis of country specifics.

It should be emphasized that the findings of Phase 2 are well-aligned with the results obtained from the users’ survey in Phase 1. Based on the study results, the EBC devised the policy recommendations for the sequence of care to optimize schizophrenia care pathways in Europe from the first episode of psychosis to long-term care [45]. The key points are summarized in Box 2.

Box 2. Summary of policy recommendations.

1. Strengthen early detection and intervention
  - Implement early psychosis intervention (EPI) programs and integrate biomarkers and AI tools for personalized treatment.
  - Address cognitive dysfunctions, negative symptoms, stress, functioning, and social challenges early to improve long-term outcomes.
2. Facilitate access to innovative treatments
  - Ensure equitable access to second-generation antipsychotics (SGAs), novel therapies, and digital health solutions.
  - Support precision medicine and clinical trials to enhance treatment effectiveness.
3. Promote coordinated care and continuity of services
  - Establish multidisciplinary teams (GPs, psychiatrists, neuro/-psychologists, social workers, public health professionals, health managers, and peers).
  - Improve care transitions between acute treatment, EIS, rehabilitation, and long-term support.
  - Address physical health risks (e.g., metabolic and cardiovascular issues in young patients) and comorbidities (e.g., addictions and cannabis use).
4. Expand access to psychosocial interventions
  - Scale evidence-based programs (CBT, cognitive remediation, psychoeducation, family therapy, vocational training, and physical exercise).
  - Integrate virtual reality therapy and digital mental health solutions to improve engagement.
5. Address social and environmental determinants, including the exposome and their impact on mental health and cognitive development
  - Develop policies for housing, financial stability, education, and employment.
  - Mitigate social factors like poverty, violence, and discrimination affecting mental health.
6. Focus on recovery and rehabilitation
  - Shift from symptom management to recovery-oriented care, prioritizing quality of life, functioning, and social inclusion.
  - Leverage digital tools to monitor progress and support patient engagement.
7. Enhance workforce capacity and training
  - Train healthcare professionals in the biopsychosocial model and digital tools, and alternatives to coercive practices.
  - Educate on emerging therapies and personalized medicine.
8. Invest in research and data-driven decision-making
  - Support longitudinal studies on integrated care models.
  - Use AI and precision medicine to optimize treatment pathways and improve outcomes.

## Conclusions

Insights from mental health professionals across Europe reveal critical challenges and opportunities in addressing stigma, enhancing effective psychosocial interventions, improving accessibility to EIS, and optimizing care pathways for schizophrenia. These findings provide actionable directions to improve outcomes for individuals with schizophrenia, supported by the latest research and clinical recommendations.

Integrating care pathways, addressing systemic disparities, and adopting patient-centered approaches are crucial to achieving equitable, high-quality care across Europe. This strategy emphasizes early intervention, fair access to treatments, coordinated care models, psychosocial support, and long-term recovery through a biopsychosocial framework and emerging technologies. While progress has been made in some regions, sustained efforts are needed to ensure that all individuals with schizophrenia have access to the support and resources they need for recovery.

## Supporting information

10.1192/j.eurpsy.2026.10172.sm001 Mohr et al. supplementary material 1 Mohr et al. supplementary material

10.1192/j.eurpsy.2026.10172.sm002 Mohr et al. supplementary material 2 Mohr et al. supplementary material

## Data Availability

The data that support the findings of this study are available from the European Brain Council upon reasonable request.

## Supplementary material

The supplementary material for this article can be found at http://doi.org/10.1192/j.eurpsy.2026.10172.

## Long descriptions

### Figure 7. Long description

The flowchart consists of three main sections connected by arrows from left to right.

1. Initial State (Left Circle): A large circle contains a central horizontal arrow pointing from At-risk state to F E P onset, illness characteristics. Surrounding this central path are several factors: Under-recognition, Stigma & Discrimination, Substance use, Self-reliance, Early recognition, services for at-risk population, and Family.

2. Health System (Center Box): A dotted arrow leads to a rectangular box titled Health system including status of mental health reforms. Inside, a dark header labeled Health services factors branches down into five specific sub-categories: Interpersonal Connections, Quality of Assessment and Care, Clinical and Functional Characterisation, Family Involvement, and Care Transitions.

3. Outcomes (Right Panels): Two arrows point from the central box to two distinct outcome scenarios.

* Top Panel (Shorter Pathway): Titled Integrated pharmacological treatments with psychological interventions and the support of innovative technologies. It lists positive factors: Positive Connections, Family Partnership, High Quality Assessment and Care, and Successful Transitions. A downward arrow leads to the bold text SHORTER PATHWAY.

* Bottom Panel (Longer Pathway): Titled Not integrated pharmacological treatments with psychological interventions. It lists negative factors: Negative Connections, Family Alienation, Delayed Detection, Unaddressed Substance Misuse, Inadequate Assessment, Low Quality Care, and Unsuccessful Transitions. A downward arrow leads to the bold text LONGER PATHWAY.

Navigate back to Figure 7..
