# Supplementary material for: Rethinking schizophrenia: Optimizing care pathways in Europe from the first episode of psychosis to long-term care
Source: Eur Psychiatry. 2026 Feb 16;69(1):e93. doi: 10.1192/j.eurpsy.2026.10172 (PMC13735790; doi:10.1192/j.eurpsy.2026.10172)
Supplement: Supplementary file 1 [file S0924933826101722sup002.docx]

*Table 1. Socio-demographic and professional characteristics of participants, by country*

| Domain | Category | Belgium | Denmark | France | Germany | Hungary | Italy | Poland | Spain | United Kingdom | Total |
| --- | --- | --- | --- | --- | --- | --- | --- | --- | --- | --- | --- |
| N (respondents) |  | 24 | 7 | 46 | 5 | 35 | 28 | 19 | 22 | 10 | 196 |
| Age (years) | <35 | 7 (29.2%) | 0 (0.0%) | 11 (23.9%) | 1 (20.0%) | 9 (25.7%) | 3 (10.7%) | 6 (31.6%) | 4 (18.2%) | 5 (50.0%) | 46 (23.5%) |
| Age (years) | 35-45 | 8 (33.3%) | 1 (14.3%) | 13 (28.3%) | 1 (20.0%) | 10 (28.6%) | 5 (17.9%) | 4 (21.1%) | 7 (31.8%) | 0 (0.0%) | 49 (25.0%) |
| Age (years) | 46-55 | 5 (20.8%) | 1 (14.3%) | 7 (15.2%) | 1 (20.0%) | 5 (14.3%) | 6 (21.4%) | 3 (15.8%) | 7 (31.8%) | 2 (20.0%) | 37 (18.9%) |
| Age (years) | 56-65 | 4 (16.7%) | 1 (14.3%) | 11 (23.9%) | 0 (0.0%) | 9 (25.7%) | 9 (32.1%) | 4 (21.1%) | 4 (18.2%) | 2 (20.0%) | 44 (22.4%) |
| Age (years) | >65 | 0 (0.0%) | 4 (57.1%) | 4 (8.7%) | 2 (40.0%) | 2 (5.7%) | 5 (17.9%) | 2 (10.5%) | 0 (0.0%) | 1 (10.0%) | 20 (10.2%) |
| Gender | Female | 12 (50.0%) | 4 (57.1%) | 27 (58.7%) | 1 (20.0%) | 16 (45.7%) | 16 (57.1%) | 8 (42.1%) | 10 (45.5%) | 7 (70.0%) | 101 (51.5%) |
| Gender | Male | 12 (50.0%) | 3 (42.9%) | 19 (41.3%) | 4 (80.0%) | 19 (54.3%) | 12 (42.9%) | 11 (57.9%) | 11 (50.0%) | 3 (30.0%) | 94 (48.0%) |
| Gender | Prefer not to disclose | 0 (0.0%) | 0 (0.0%) | 0 (0.0%) | 0 (0.0%) | 0 (0.0%) | 0 (0.0%) | 0 (0.0%) | 1 (4.5%) | 0 (0.0%) | 1 (0.5%) |
| Profession | Psychiatrist | 6 (25.0%) | 5 (71.4%) | 40 (87.0%) | 4 (80.0%) | 29 (82.9%) | 27 (96.4%) | 15 (78.9%) | 17 (77.3%) | 5 (50.0%) | 148 (75.5%) |
| Profession | Psychologist | 7 (29.2%) | 1 (14.3%) | 0 (0.0%) | 0 (0.0%) | 4 (11.4%) | 0 (0.0%) | 4 (21.1%) | 2 (9.1%) | 2 (20.0%) | 20 (10.2%) |
| Profession | Nurse | 5 (20.8%) | 0 (0.0%) | 2 (4.3%) | 1 (20.0%) | 2 (5.7%) | 0 (0.0%) | 0 (0.0%) | 2 (9.1%) | 3 (30.0%) | 15 (7.7%) |
| Profession | Social Worker | 3 (12.5%) | 1 (14.3%) | 0 (0.0%) | 0 (0.0%) | 0 (0.0%) | 0 (0.0%) | 0 (0.0%) | 0 (0.0%) | 0 (0.0%) | 4 (2.0%) |
| Profession | General Practitioner | 1 (4.2%) | 0 (0.0%) | 1 (2.2%) | 0 (0.0%) | 0 (0.0%) | 0 (0.0%) | 0 (0.0%) | 0 (0.0%) | 0 (0.0%) | 2 (1.0%) |
| Profession | Other (please specify) | 2 (8.3%) | 0 (0.0%) | 3 (6.5%) | 0 (0.0%) | 0 (0.0%) | 1 (3.6%) | 0 (0.0%) | 1 (4.5%) | 0 (0.0%) | 7 (3.6%) |
| Professional experience (years) | <5 | 5 (20.8%) | 0 (0.0%) | 5 (10.9%) | 1 (20.0%) | 4 (11.4%) | 2 (7.1%) | 4 (21.1%) | 2 (9.1%) | 2 (20.0%) | 25 (12.8%) |
| Professional experience (years) | 5-10 | 6 (25.0%) | 1 (14.3%) | 11 (23.9%) | 0 (0.0%) | 8 (22.9%) | 5 (17.9%) | 4 (21.1%) | 3 (13.6%) | 2 (20.0%) | 40 (20.4%) |
| Professional experience (years) | 11-25 | 8 (33.3%) | 2 (28.6%) | 14 (30.4%) | 2 (40.0%) | 10 (28.6%) | 7 (25.0%) | 4 (21.1%) | 10 (45.5%) | 3 (30.0%) | 60 (30.6%) |
| Professional experience (years) | 26-40 | 5 (20.8%) | 0 (0.0%) | 12 (26.1%) | 0 (0.0%) | 10 (28.6%) | 10 (35.7%) | 6 (31.6%) | 6 (27.3%) | 3 (30.0%) | 52 (26.5%) |
| Professional experience (years) | >40 | 0 (0.0%) | 4 (57.1%) | 4 (8.7%) | 2 (40.0%) | 3 (8.6%) | 4 (14.3%) | 1 (5.3%) | 1 (4.5%) | 0 (0.0%) | 19 (9.7%) |

**Note:** Percentages are calculated within each country.
